# Measurement of supportive attitudes towards intimate partner violence against women among a Spanish-speaker sample

**DOI:** 10.1371/journal.pone.0241392

**Published:** 2020-11-03

**Authors:** Andrés Sánchez-Prada, Carmen Delgado-Alvarez, Esperanza Bosch-Fiol, Virginia Ferreiro-Basurto, Victoria A. Ferrer-Perez

**Affiliations:** 1 Faculty of Psychology, Pontifical University of Salamanca, Salamanca, Spain; 2 Faculty of Psychology, University of Balearic Islands, Palma, Spain; Murcia University, Spain, SPAIN

## Abstract

Intimate partner violence is the most common type of violence against women. Attitudes towards this violence are increasingly recognized as key to understanding this social and public health problem because a social environment that accepts or even supports it creates a climate that breeds further violence and encourages their perpetration. The evidence available shows that these attitudes are influenced by different individual, organizational and community factors, and that the supportive attitudes are generally more common among males, and among older and less educated people. This paper presents two cross-sectional studies which aim to obtain a deeper understanding of supportive attitudes towards intimate partner violence against women in a Spanish-speakers context. Results obtained show that the two questionnaires used may be useful for evaluating supporting attitudes towards this violence in Spanish-speaking samples. Thus, the Inventory of Distorted Thoughts about Women and Violence (IPDMV), one of the one of the most widely used tools to this aim among Spanish-speakers, includes information regarding the minimization of this violence and the responsibility of perpetrators, and it seems better able to capture the effect of previous training, which would be consistent with the fact that it was initially designed to detect the effects of interventions; and the Inventory of Beliefs about Intimate Partner Violence (IBIPV), a new tool recently designed to this aim, is more focused on supportive attitudes and seems more effective for capturing differences between men and women in blaming victims and exonerating perpetrators. Additionally, the results obtained allow us to complement previous studies on the effects that factors such as gender, age, or previous training have on supportive attitudes towards intimate partner violence against women.

## Introduction

About one third of all women globally will experience either intimate partner or non-partner physical or sexual violence during their lifetime. Intimate partner violence is the most common type of violence against women (VAW), affecting 30% of women worldwide, according the World Health Organization [1–5] and the Fundamental Right Agency [6] analysis.

It is important to remember that the Declaration on the Elimination of Violence against Women [7] provided a definition for VAW that has been adopted as a benchmark for most of the international bodies that contend with this matter. More recently, the Council of Europe Convention on preventing and combating violence against women and domestic violence [8] continues in this vein and understands VAW as a form of gender-based violence that is committed against women because they are women, defining VAW in its 3^rd^ article “*as a violation of human rights and a form of discrimination against women and shall mean all acts of gender-based violence that result in*, *or are likely to result in*, *physical*, *sexual*, *psychological or economic harm or suffering to women*, *including threats of such acts*, *coercion or arbitrary deprivation of liberty*, *whether occurring in public or in private life*” (p. 3). They understand intimate partner violence to mean all acts of physical aggression, sexual coercion, psychological abuse or economic violence between current or former spouses or partners, which affect women disproportionately and which are therefore distinctly gendered [9] and usually referred to as intimate partner violence against women (IPVAW).

In this context, the need for all sectors to engage in addressing and eliminating attitudes, norms and beliefs that tolerate and justify VAW and IPVAW, and to offer better support for women who experience these forms of violence is crucial in strategies intended to prevent, reduce and eradicate VAW and IPVAW, as pointed out by different sources [5, 10–12]. Thus, the social consideration of VAW and IPVAW is particularly important because “*prevalence rates for any form of family violence are related to attitudes that predominate in a society*” [13, p. 763] and “*an understanding of attitudes towards VAW is vital for effective prevention strategies*” [14, p. 333]. In particular, “*attitudes held by societies at large can influence responses to violence*, *such as availability of refuge for victims*, *criminalization of violence*, *and victims’ interpretations of and recovery from their experience (e*.*g*. *shame and guilt experienced)*” [14, p. 334], and “*challenging and changing attitudes that tolerate and justify IPVAW has become a key objective for intervention and prevention initiatives addressing IPVAW*” [15, p. 2]. In summary, attitudes towards IPVAW are increasingly recognized as key to understanding this social and public health problem because a social environment that accepts or even supports VAW and IPVAW creates a climate that breeds further violence and encourages their perpetration, making it easier for perpetrators to persist in their violent behavior, and making it more difficult for women as well as the general population to react against IPVAW [11, 12, 15–25].

The attitudes supporting this violence may be characterized as [15, 19, 26]: acceptability of IPVAW (including acceptance, approval, tolerance, and permissively of this violence); minimization of the importance of IPVAW; and legitimation of IPVAW (victim blaming, legitimation, and justification or exoneration of the perpetrator). A cross-cultural analysis shows that there are some regional and cultural differences in supportive attitudes towards IPVAW [5, 14, 23, 25, 27–31]; therefore, we will focus on a review of these attitudes in Europe and in particular Spain, where the studies presented in this paper are developed.

Relating to the acceptability of VAW and IPVAW, some general surveys carried out in the European Union [32, 33] showed that the majority of the people considered IPVAW as unacceptable in all circumstances and always punishable by law, with this rejection gaining ground over time; only about 1–2% of the people interviewed thought this violence as acceptable in certain circumstances or even in all circumstances. In line with these findings, Tausch [30] analyzed data from the World Values Survey project, and observed that some Western European countries (such as Italy or Norway) are among those with the lowest rates of acceptance of IPVAW. However, it is important to remark that, despite this rejection, European Union surveys [11, 29, 33, 34] showed a high prevalence of IPVAW victim blaming attitudes in the EU, and the number of people who consider the provocative behavior of women as a cause of such violence actually increased between these two surveys, from 46.1% in 1999 to 52% in 2010.

Additionally, Waltermaurer [35] conducted a systematic review, based mainly on results from national surveys, and pointed out that “*there are many gaps to our knowledge internationally about the justification of IPV*, *particularly in Europe and the Western Hemisphere*” (p. 173), concluding that research on attitudes towards IPVAW remain underdeveloped. Subsequently, Gracia and Lila [12] conducted a non-systematic review of national surveys on attitudes towards IPVAW yielded in the EU Member States between 2010 and 2014 and found that only a relatively limited number of the surveys (forty in nineteen countries) included questions addressing attitudes towards VAW or IPVAW), most of which were either based on a single item or non-supported by instruments with enough evidence of validity and reliability. Their review concludes that a small but relevant percentage of respondents from different countries tended to ‘accept’ in some circumstances some violent behaviors against women, perceived as ‘not very serious’ or considered ‘inevitable’, and also that victim-blaming attitudes were not widespread. More recently, Gracia et al. [15] developed a systematic review that included 62 quantitative studies addressing attitudes towards IPVAW conducted in the EU between 2000 and 2018, and provided a conceptual map of attitudes towards IPVAW, as well a map of approaches to measuring these attitudes used in Europe.

In Spain, the data available from surveys show that the social rejection of this violence reaches rates around 90–95% [36–39], and victim blaming attitudes reaches levels around 30–35% [11, 29, 33, 40].

As reflected some literature reviews [19, 25, 30], the evidence available shows that attitudes towards VAW and IPVAW are influenced by different individual, organizational and community factors, and that one of the strongest predictors of supportive attitudes towards IPVAW is gender [15, 19, 30, 41–43].

Although there were some differences across countries [25, 30], a general gender gap [19, 23, 26, 27, 29, 31, 43] from an early age [44] has been identified. Specifically, in Europe, general surveys [32–34], studies with student samples [45–47] and with general populations [15, 40, 43, 46, 48–51] found that men are more likely to justify and accept this violence, to perceive a narrower range of behaviors as violent, to minimize the assaults and see violent behaviors against women as less serious, inappropriate, or damaging, and to exonerate the perpetrator and blame women victims for the violence experienced; while women are more likely to reject this violence, to attribute the violence responsibility to the perpetrator and to consider violent incidents more serious. The results obtained would lead one to reasonably conclude that these differences probably do not reflect sex differences, but ideological differences based in gender norms and social ideologies about male domination over women [14, 19, 51, 52].

The respondent’s age, and the developmental processes associated with it, also seem to be a factor in shaping attitudes towards IPVAW [19, 25], but the results are inconsistent [15, 27, 43, 46, 50]. Indeed, some studies show older people are more likely to accept IPVAW as normal and justifiable, and to legitimize it [26, 29, 40, 52, 53], while other studies suggest that attitudes justifying IPVAW are more likely among the youngest age groups [35].

Regarding the respondent’s education, studies carried out in high income countries find a negative relationship between educational level and attitudes supporting IPVAW, such as acceptance or victim blaming, which are more common among less educated respondents [23, 40].

In summary, as Gracia et al. [12, 15] point out after reviewing European research, that supportive attitudes towards IPVAW were generally more common among males, and among older and less educated people.

The importance of these beliefs and attitudes towards IPVAW makes it necessary to have reliable and valid measures for research and intervention purposes [54, 55]. Although a multitude of evaluation instruments have been designed for this purpose [56], some of them have not yielded sufficient information supporting their latent structure or dimensionality or have lacked sufficient psychometric support for both internal and external construct validity [15].

In a Spanish context, one of the most widely used tools for this purpose is the Inventory of Distorted Thoughts about Women and Violence (IPDMV, in the Spanish acronym). This questionnaire was designed by Echeburúa and Fernández-Montalvo [57, 58] for clinical purposes as a part of a cognitive-behavioural programme for treating batterers. Originally it was a checklist of 29 binary items about irrational thoughts related to traditional gender roles and to the use of violence as an acceptable strategy for solving conflicts. Subsequent studies carried on in different contexts and with different kinds of samples [59–63] obtained different factorial models (for a review of these studies and models see [61]). Some of these studies have found differences by gender in beliefs and attitudes about IPVAW [16, 60, 61, 63], and also by other variables such as specific academic-training on IPV.

More recently, Garcia-Ael et al. [64] developed the Inventory of Beliefs about Intimate Partner Violence (IBIPV), a self-report scale designed to assess participants’ attitudes towards wife beating. This tool is a revised and up-dated version of the Inventory of Beliefs about Wife Beating (IBWB, [65]) that takes the latest theoretical and empirical advances in the IPVAW field into account. When developing the IBIPV, the authors eliminated some items from IBWB (specifically, those having nothing to do with beliefs about IPVAW, those linked to a masochistic image of the woman, or any that were repeated). Additionally, they eliminated items referring to the same concept or those considered too generic, and revised and adapted the terminology used in the original survey in order to bring the questions more in line with the current conception of IPVAW (i.e. replacing ‘husband’ with ‘partner’ or ‘abuser’, or ‘wife’ by ‘partner’ or ‘woman’). Nine new items were added to cover some theoretical and empirical aspects not addressed by the original inventory. The result is a new 22-items instrument, with three subscales: Justifying Partner Violence (JPV), a subscale related to behaviors of victims and perpetrators which may be used to legitimize or justify IPVAW; Victims Responsible for Violence, a subscale (VRV) related to victim behaviors which may lead to women being blamed for IPVAW; and Abuser Responsible for Violence, a subscale (ARV) related to the behaviors of perpetrators, which may lead to consider them as being responsible for IPVAW. The results obtained by their authors showed that IBIPV has good psychometric properties and the potential to be a tool for measuring attitudes to IPVAW. Additionally, they found significant age and gender-related differences in scores for some of their subscales.

This paper presents two cross-sectional studies undertaken sequentially, which aim to obtain a deeper understanding of supportive attitudes towards IPVAW, once reviewed some basic psychometric properties of two measurement instruments used.

## Study 1

The aim of Study 1 is to review the internal consistency and factorial structure of IPDMV (one of the most used tools to analyze attitudes towards IPVAW in Spain) and the IBIPV (an adaptation for the Spanish population of the IBWB, a classic tool to analyze these attitudes), as well as to examine the correlations between their subscales in order to establish the level of convergence-divergence of both instruments.

### Materials and methods

The research protocol for this study was approved by the Bioethics Committee of the University of Balearic Islands (Ref. 5487, 23th july 2015). The participants completed a written consent form.

#### Participants

An opportunity sample comprising 1,132 IBIPV cases (of which 30.4% were against men and 69.3% against women) and 2,114 IPDMV cases (of which 31.6% were against men and 67.0% against women) was used. Participants were Spanish volunteers recruited in 2018–2019 by announcements in different Spanish education centers (participating universities, vocational training centers and high schools). The average age of the IBIPV cases was *M* = 24.15 years (*SD* = 11.85), and in the IPDMV cases it was *M* = 23.16 (*SD* = 8.62).

#### Instruments

The **Inventory of Beliefs about Intimate Partner Violence** (IBIPV, [64]) comprises 22 items rated on a seven-point scale from 1 (*totally disagree*) to 7 (*totally agree*) which reflect three dimensions: *Justifying Partner Violence* (F1-JPV, 6-items, α = .71); *Victims Responsible for Violence* (F2-VRV, 9-ítems, α = .93); and *Abuser Responsible for Violence* (F3-ARV, 7-items, α = .84). After reversing the scores of item 3, and the seven items of subscale ARV, so that all 22 items maintain the same sense in the construct, higher scores indicate higher tolerance towards IPVAW (i.e. lower rejection towards IPVAW). The **Inventory of Distorted Thoughts about Women and Violence** (IPDMV in the Spanish acronym, [57, 58]; adapted version of Ferrer et al. [61]) comprises 24 items related to four dimensions: *Inferiority of Women Compared to Men* (F1-IW, 7 items, α = .86); *Blaming Female Victims of Abuse* (F2-BW, 7 items, α = .62); *Violence as an Appropriate Problem-solving Strategy* (F3-VP, 5 items: α = .69); and *Minimization and Exoneration of the Abuser* (F4-MA, 5 items, α = .53). Responses are given in a four-point scale from 1 (*totally disagree*) to 4 (*totally agree*), and the higher the scores, the higher the levels of distorted thoughts (i.e. the lower the rejection towards IPVAW).

Finally, participants completed a brief questionnaire designed ad hoc, including some sociodemographic data, such as gender and age, and some questions about their previous training in IPVAW.

#### Procedure

The participants read over the consent form and those who voluntarily agreed completed a brief questionnaire on sociodemographic data, followed by the IPDMV and the IBIPV. The questionnaires were mainly completed on a computer, adapted to paper-and-pencil format for those who required it.

#### Data analysis

Analyses were carried out with IBM SPSS 25 and AMOS 23. Corrected homogeneity indices and Cronbach’s alpha coefficients were used to analyze internal consistency, and Confirmatory Factor Analysis (CFA) was employed to test the factorial structure of both instruments. Corrected homogeneity indices and Cronbach’s alpha plus McDonald’s omega coefficients were used. The Unweighted Least Squares estimation method (ULS) was chosen for CFA, since it is recommended for ordinal variables [66, 67], and multiple criteria were selected for fit testing [68–70], namely: the Goodness of Fit Index (GFI ≥ .95), the Adjusted Goodness of Fit Index (AGFI ≥ .90), the Normed Fit Index (NFI ≥ .95), and the Standardized Root Mean Square Residual (SRMR ≤ .05). We also took into consideration two parsimonious fit indexes, the PGFI and the PNFI, for which a generally accepted value is around .50 if models show a good fit in all other indices [71]. To analyze convergence and divergence between the IBIPV and IPDMV subscales we used Pearson’s correlation coefficients.

### Results

#### Instruments’ reliability and factorial structure of IBIPV and IPDMV

**Fig 1:**
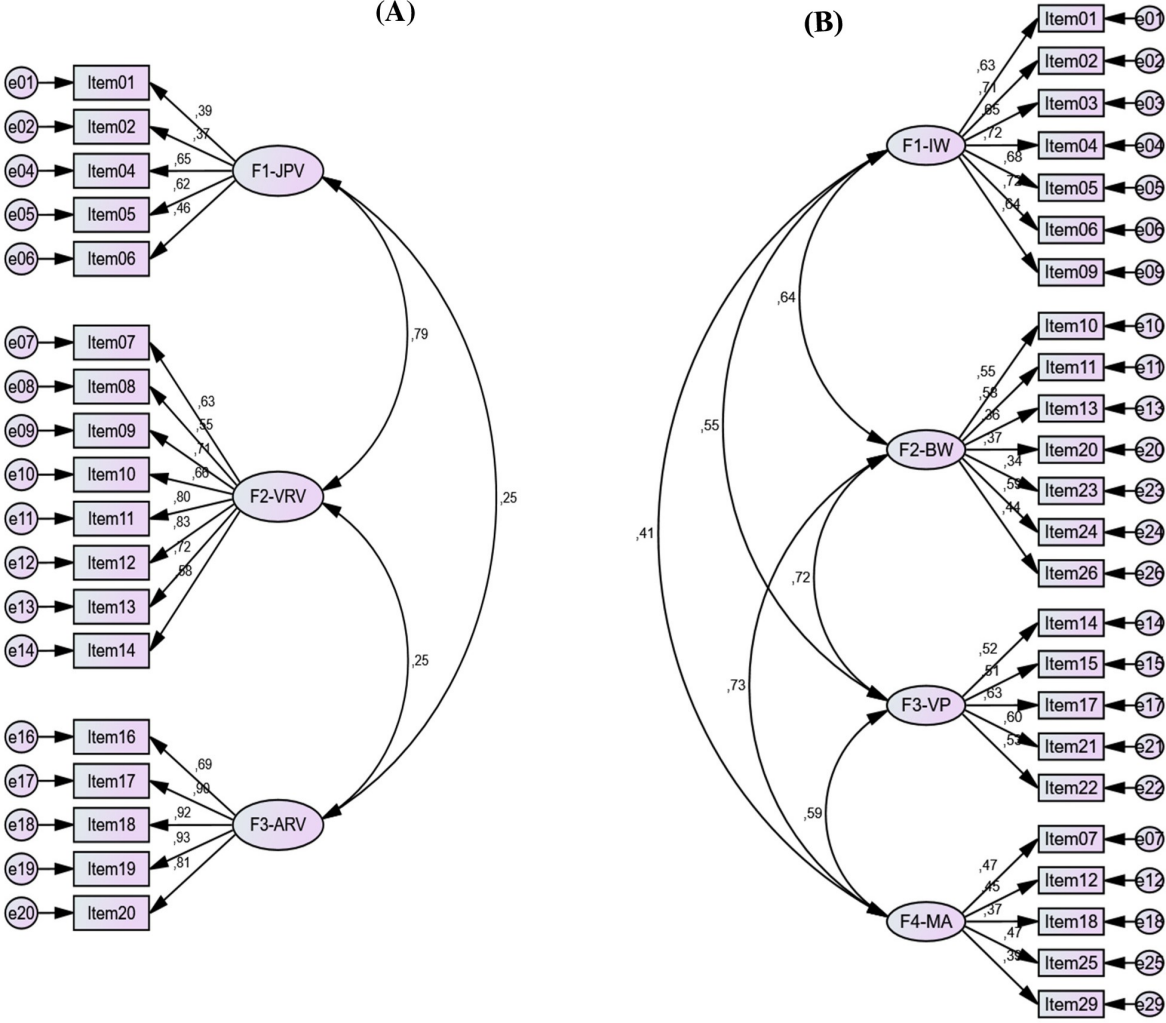
Factor loadings and correlations between factors for the IBIPV and the IPDMV. (A) IBIPV: F1-JPV: Justifying Partner Violence; F2-VRV: Victims Responsible for Violence; F3-ARV: Abuser Responsible for Violence (this dimension’s items are inverted so that it is expressed in the opposite sense, that is, “Abuser not Responsible for Violence”). (B) IPDMV: F1-IW: Inferiority of Women Compared to Men; F2-BW: Blaming Female Victims of Abuse; F3-VP: Violence as an Appropriate Problem-solving Strategy; F4-MA: Minimization and Exoneration of the Abuser.

The internal consistency of the IBIPV was satisfactory for the overall scale (α = .79), as well as for the subscales F2-VRV (α = .77, ω = .84) and F3-ARV (α = .82, ω = .87), but quite lower for the subscale F1-JPV (α = .41, ω = .33). The subsequent CFA leads us to revise the model, since the SRMR indicated a not good enough fit (Table 1). Based on the analysis of the corrected homogeneity indices (HIc), items that negatively affected the internal consistency were removed; that is, item 03 (HIc = .103 in the subscale F1-JPV), item 15 (HIc = .296 in the subscale F2-VRV), and items 21 and 22 (HIc = .044 and HIc = .296, respectively, in the subscale F3-ARV). As can be observed in Table 1, the fit of the revised model with 18 items was very satisfactory, and reliability improved for the overall scale (α = .84) and for each of the subscales (F1-JPV: α = .64, ω = .63; F2-VRV: α = .86, ω = .88; F3-ARV: α = .93, ω = .93). Accordingly, this 18-item model was taken as reference for further analysis, in spite of the relatively lower internal consistence of the subscale F1-JPV. The proposed model’s factor structure is displayed in Fig 1.

**Table 1:** Goodness-of-fit indexes of the IBIPV and the IPDMV.

Index	Good fit criterion	IBIPV original model 3 factors; 22 items	IBIPV revised model 3 factors; 18 items	IPDMV original model 4 factors; 24 items
**GFI**	≥ .95	.987	.998	.984
**AGFI**	≥ .90	.984	.997	.980
**PGFI**	≥ .50	.804	.770	.807
**NFI**	≥ .95	.976	.997	.953
**PNFI**	≥ .50	.871	.860	.849
**SRMR**	≤ .05	.0987	.0469	.0496

GFI: Goodness-of-Fit Index; AGFI: Adjusted Goodness-of-Fit Index; PGFI: Parsimony Goodness-of-Fit Index; NFI: Normed Fit Index; PNFI: Parsimony Normed Fit Index; SRMR: Standardized Root Mean Square Residual.

Regarding the IPDMV, Cronbach’s alpha coefficients were adequate for the overall scale (α = .82) and for subscale F1-IW (α = .86, ω = .86), but lower for subscales F2-BW (α = .62, ω = .66), F3-VP (α = .68, ω = .69), and especially for subscale F4-MA (α = .53, ω = .53). However, the CFA showed a satisfactory model’s fit (Table 1, Fig 1). These results, in addition to the corrected homogeneity indices, which indicated a positive contribution of all the IPDMV-items to their respective subscale’s internal consistency, lead us to maintain the actual model for further analysis, despite the lower reliability of some of their subscales.

#### Relationships between IBIPV and IPDMV

As shown in Table 2, all of the subscales of the IBIPV and the IPDMV were positively and significantly correlated, except the F3-ARV IBIPV subscale and the F4-MA IPDMV subscale. The stronger correlations were found between the F1-JPV and F2-VRV subscales of the IBIPV on the one hand, and between the F1-IW and F2-BW subscales of the IPDMV on the other hand (from .37 to .55), which reflects a moderate-high association between attitudes that justify IPVAW, the former assert women inferiority while the latter blame them for the violence suffered. These four subscales showed low-moderate correlations with the subscales of the other instrument related to men’s responsibility (F3-ARV IBIPV subscale and F4-MA IPDMV subscale; from .21 to .26). Finally, the correlations between the F3-VP subscale of the IPDMV and the IBIPV subscales ranged from low to moderate (.13 to .28). These convergences and divergences between instruments were taken into account in the forthcoming analysis.

**Table 2:** Correlations between IBIPV and IPDMV subscales.

		IPDMV			
		F1-IW	F2-BW	F3-VP	F4-MA
**IBIPV**	**F1-JPV**	.491***	.369***	.254***	.213***
	**F2-VRV**	.546***	.434***	.279***	.233***
	**F3-ARV**^**a**^	.223***	.259***	.135***	.032

IBIPV: F1-JPV: *Justifying Partner Violence*; F2-VRV: *Victims Responsible for Violence*; F3-ARV: *Abuser Responsible for Violence*

IPDMV: F1-IW: *Inferiority of Women Compared to Men*; F2-BW: *Blaming Female Victims of Abuse*; F3-VP: V*iolence as an Appropriate Problem-solving Strategy*; F4-MA: *Minimization and Exoneration of the Abuser*

^a^ Item scores are inverted. The dimension is expressed in the opposite sense (*not responsible*).

*** *p* < .001.

## Study 2

The aim of Study 2 is to analyze the attitudes towards IPVAW across dimensions, and to compare them by gender, age, and specific training in IPVAW.

### Materials and methods

The research protocol for this study was approved by the Bioethics Committee of the University of Balearic Islands (Ref. 5487, 23th july 2015). The participants completed a written consent form.

#### Participants

Participants that answered both the IBIPV and IPDMV (n = 901) were included in the sample of the Study 2. This sample was composed of 285 men (31.6%), 613 women (68.0%) and three people identified as other options (0.3%), ranging from 16 to 82 years (*M =* 24.18; *SD =* 13.38). The majority (97.4*%*, *n* = 878) were students at the moment of participation: of these, 614 were at the university (69.9%), 123 were studying vocational training (14.0%) and 141 were high school students (16.1%). Related to the previous academic-training in IPVAW, 46.8% of the sample (*n* = 422) had studied some topic on this issue, and 48.9% (*n* = 441) had participated in at least one non-curricular activity. There were no differences between women and men samples regarding age (*t* (454.995) = 1.219; *p* = .223) and participation in non-curricular activities (χ^2^ (1) = 3.740; *p* = .053). However, more women (50.6%) than men (40.9%) had studied some topic specifically related to IPVAW (χ^2^ (1) = 7.174; *p* = .007).

#### Instruments

For this study they were used the same instruments as for Study 1, that is: the **Inventory of Beliefs about Intimate Partner Violence** (IBIPV, [64]), the **Inventory of Distorted Thoughts about Women and Violence** (IPDMV [57, 58]; adapted version of Ferrer et al. [61]); and a brief questionnaire designed ad hoc, including some sociodemographic data.

#### Procedure

The procedure was also similar to the Study 1.

#### Data analysis

Analyses were carried out with IBM SPSS 25 and AMOS 23. In order to analyze the attitudes towards IPVAW across dimensions. Given the different sample sizes and non-homoscedasticity across groups, we opted for nonparametric (Mann-Whitney and Kruskal-Wallis) tests to compare these attitudes by gender, age, and specific training in IPVAW.

### Results

#### Differences between attitude dimensions by gender

**Fig 2:**
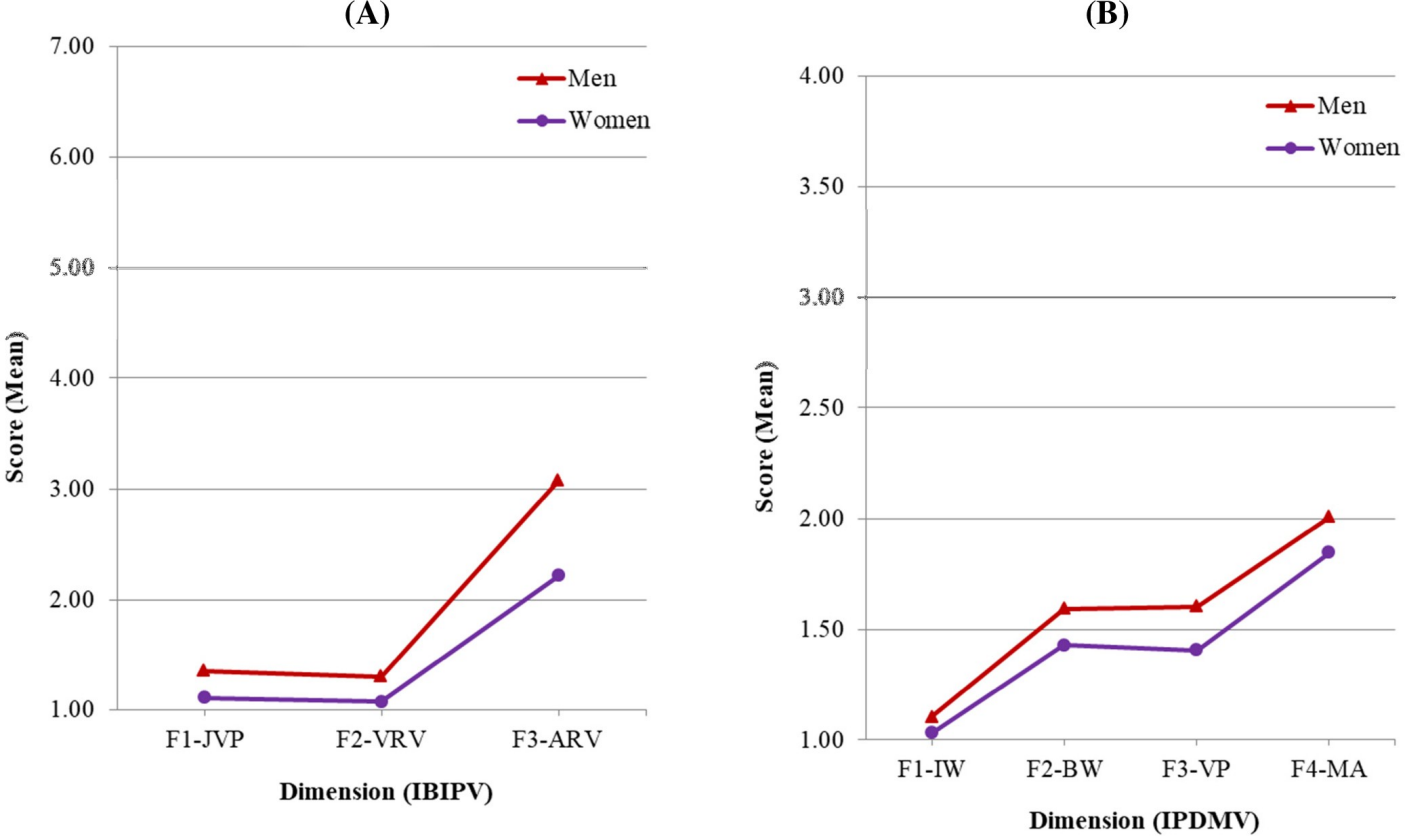
Mean scores of attitude dimensions by gender. (A) IBIPV: F1-JPV: Justifying Partner Violence; F2-VRV: Victims Responsible for Violence; F3-ARV: Abuser Responsible for Violence. (B) IPDMV: F1-IW: Inferiority of Women Compared to Men; F2-BW: Blaming Female Victims of Abuse; F3-VP: Violence as an Appropriate Problem-solving Strategy; F4-MA: Minimization and Exoneration of the Abuser.

Mean scores of IBIPV and IPDMV subscales are displayed in Table 3 and Fig 2, where a similar pattern can be observed for women and men samples. Regarding the IBIPV, both women and men showed a stronger rejection towards IPVAW in F1-JPV (*M*_men_ = 1.35; *M*_women_ = 1.11) and in F2-VRV (*M*_men_ = 1.31; *M*_women_ = 1.07), compared to the F3-ARV subscale (*M*_men_ = 3.08; *M*_women_ = 2.22). In IPDMV both subsamples showed similar scores in F2-BW (*M*_men_ = 1.59; *M*_women_ = 1.43) and F3-VP (*M*_men_ = 1.60; *M*_women_ = 1.41) subscales (*p* = 1.0). In the same vein, the highest level of rejection towards IPVAW was found in F1-IW (*M*_men_ = 1.10; *M*_women_ = 1.03), and the lowest in F4-MA (*M*_men_ = 2.01; *M*_women_ = 1.85).

**Table 3:** Differences in attitudes dimensions by gender.

Subscale	Gender (*n*)	*M* (*SD*)	Mean Rank	*U*	*Z*	*p*
**IBIPV**						
**F1-JVP**	**Men (*n* = 277)**	1.35 (0.67)	523.04	60374.000	-8.929	< .001
	**Women (*n* = 602)**	1.11 (0.42)	401.79			
**F2-VRV**	**Men (*n* = 275)**	1.31 (0.64)	520.93	60244.000	-8.480	< .001
	**Women (*n* = 602)**	1.07 (0.31)	401.57			
**F3-ARV**	**Men (*n* = 266)**	3.08 (1.91)	511.37	56959.000	-6.545	< .001
	**Women (*n* = 592)**	2.22 (1.51)	392.71			
**IPDMV**						
**F1-IW**	**Men (*n* = 280)**	1.10 (0.27)	502.62	69125.500	-7.019	< .001
	**Women (*n* = 609)**	1.03 (0.12)	418.51			
**F2-BW**	**Men (*n* = 271)**	1.59 (0.43)	492.59	62171.000	-5.161	< .001
	**Women (*n* = 586)**	1.43 (0.32)	399.59			
**F3-VP**	**Men (*n* = 275)**	1.60 (0.57)	496.76	66891.000	-4.647	< .001
	**Women (*n* = 602)**	1.41 (0.42)	412.61			
**F4-MA**	**Men (*n* = 270)**	2.01 (0.61)	475.04	67894.000	-3.534	< .001
	**Women (*n* = 591)**	1.85 (0.53)	410.88			

IBIPV: F1-JPV: *Justifying Partner Violence*; F2-VRV: *Victims Responsible for Violence*; F3-ARV: *Abuser Responsible for Violence*

IPDMV: F1-IW: *Inferiority of Women Compared to Men*; F2-BW: *Blaming Female Victims of Abuse*; F3-VP: V*iolence as an Appropriate Problem-solving Strategy*; F4-MA: *Minimization and Exoneration of the Abuser*

In order to analyze differences by gender in each of the subscales, a series of subsequent Mann-Whitney tests were conducted, adjusting the levels of significance to control type I errors. As observed also in Fig 2 and in Table 3, there were significant differences by gender in all of the subscales in both IBIPV and IPDMV, and in both cases: women expressed a stronger rejection towards IPVAW than men.

#### Age differences in attitude dimensions

Four age ranges were established in the analysis of differences by age (16 to 17 years; 18 to 24 years; 25 to 49 years; and 50 to 82 years). Due to the heterogeneous size of age groups, we undertook a nonparametric Kruskal-Wallis test to compare each subscale’s scores across them. These are displayed in Table 4.

**Table 4:** Differences in attitude dimensions by age.

Subscale	Age range (*n*)	*M* (*SD*)	Mean Rank	*H (df = 3)*	*p*
**IBIPV**					
**F1-JVP**	**16–17 (*n* = 110)**	1.40 (0.70)	540.47	60.065	**< .001**
	**18–24 (*n* = 611)**	1.13 (0.40)	417.80		
	**25–49 (*n* = 86)**	1.08 (0.30)	391.79		
	**50–82 (*n* = 70)**	1.49 (0.92)	522.56		
**F2-VRV**	**16–17 (*n* = 104)**	1.32 (0.59)	536.41	38.272	**< .001**
	**18–24 (*n* = 616)**	1.10 (0.33)	421.49		
	**25–49 (*n* = 86)**	1.08 (0.22)	404.70		
	**50–82 (*n* = 70)**	1.38 (0.98)	484.23		
**F3-ARV**	**16–17 (*n* = 104)**	3.26 (1.77)	549.53	28.991	**< .001**
	**18–24 (*n* = 606)**	2.32 (1.59)	410.24		
	**25–49 (*n* = 83)**	2.42 (1.61)	434.36		
	**50–82 (*n* = 65)**	2.75 (2.18)	410.78		
**IPDMV**					
**F1-IW**	**16–17 (*n* = 113)**	1.11 (0.24)	513.15	45.787	**< .001**
	**18–24 (*n* = 617)**	1.04 (0.18)	429.50		
	**25–49 (*n* = 86)**	1.01 (0.05)	396.70		
	**50–82 (*n* = 71)**	1.11 (0.20)	517.27		
**F2-BW**	**16–17 (*n* = 107)**	1.63 (0.42)	524.45	29.934	**< .001**
	**18–24 (*n* = 603)**	1.46 (0.35)	415.77		
	**25–49 (*n* = 85)**	1.36 (0.29)	355.45		
	**50–82 (*n* = 62)**	1.60 (0.45)	493.81		
**F3-VP**	**16–17 (*n* = 110)**	1.66 (0.55)	537.31	40.471	**< .001**
	**18–24 (*n* = 611)**	1.45 (0.46)	433.16		
	**25–49 (*n* = 87)**	1.26 (0.37)	316.24		
	**50–82 (*n* = 67)**	1.54 (0.51)	477.24		
**F4-MA**	**16–17 (*n* = 105)**	2.20 (0.60)	555.25	48.799	**< .001**
	**18–24 (*n* = 606)**	1.83 (0.52)	409.43		
	**25–49 (*n* = 86)**	1.73 (0.52)	355.71		
	**50–82 (*n* = 63)**	2.15 (0.69)	527.31		

IBIPV: F1-JPV: *Justifying Partner Violence*; F2-VRV: *Victims Responsible for Violence*; F3-ARV: *Abuser Responsible for Violence*

IPDMV: F1-IW: *Inferiority of Women Compared to Men*; F2-BW: *Blaming Female Victims of Abuse*; F3-VP: V*iolence as an Appropriate Problem-solving Strategy*; F4-MA: *Minimization and Exoneration of the Abuser*

**Fig 3:**
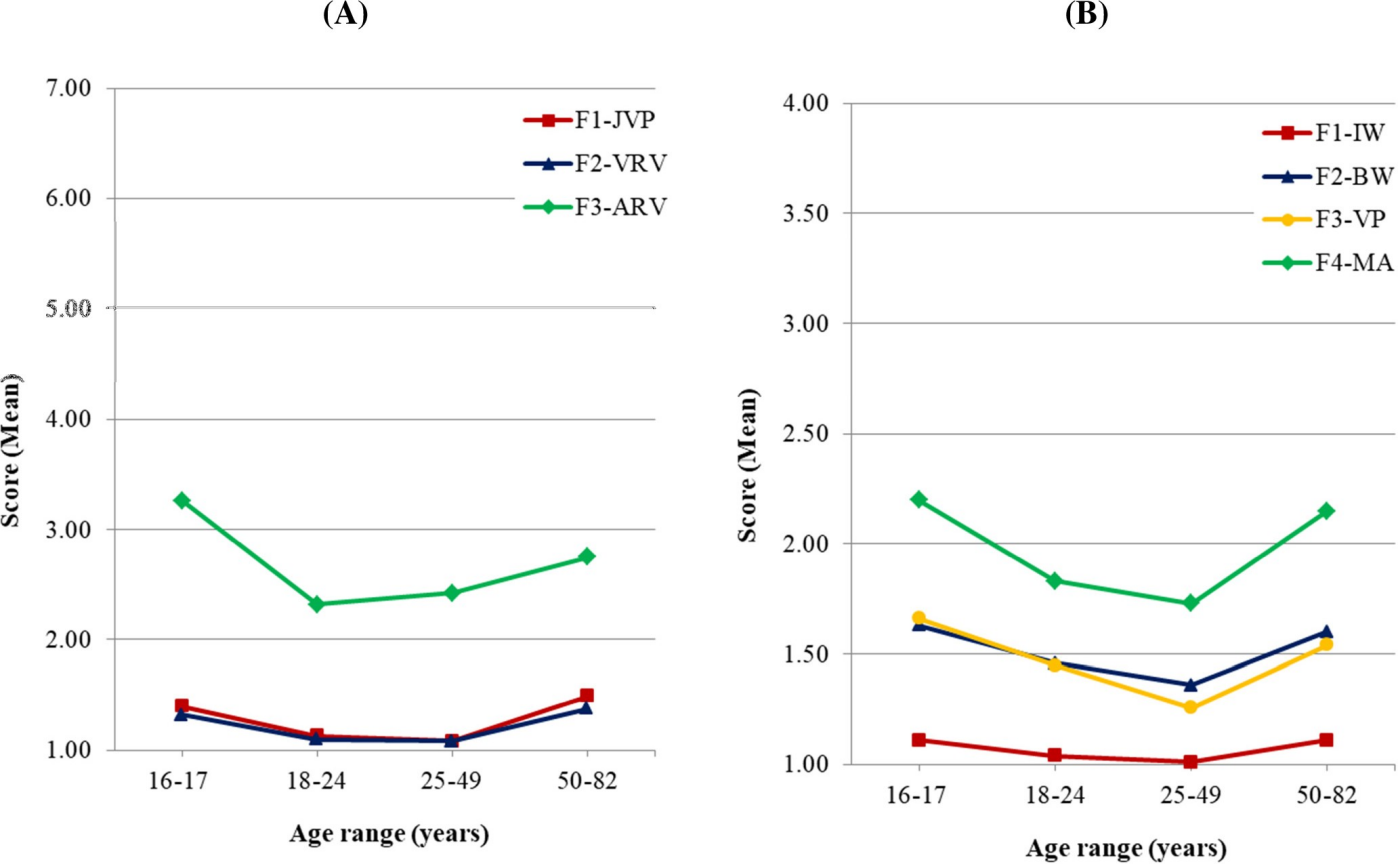
Mean scores of attitude dimensions by age group. (A) IBIPV: F1-JPV: Justifying Partner Violence; F2-VRV: Victims Responsible for Violence; F3-ARV: Abuser Responsible for Violence. (B) IPDMV: F1-IW: Inferiority of Women Compared to Men; F2-BW: Blaming Female Victims of Abuse; F3-VP: Violence as an Appropriate Problem-solving Strategy; F4-MA: Minimization and Exoneration of the Abuser.

The Kruskal-Wallis test yielded statistically significant age effects on all of the subscales of both IBIPV and IPDMV. Pairwise comparisons with Bonferroni correction showed the following differences between age groups: First, in the F1-JPV IBIPV subscale, as well as the F1-IW and F4-MA IPDMV subscales, a lower rejection towards IPVAW was observed in the 16–17 and 50–82 age groups, compared with the 18–24 and 25–49 age groups (*p* < .001 in all comparisons, except between 18–24 and 50–82 in F4-MA, *p* = .002). Second, in the F3-ARV IBIPV subscale the rejection in the 16–17 age group was lower than in the 18–24 (*p* < .001), the 25–49 (*p* = .009) and the 50–82 age groups (*p* = .002). Third, in the F2-VRV IBIPV subscale and F2-BW IPDMV subscale the 16–17 group showed less rejection than the 18–24 and the 25–49 age groups (*p* < .001). Finally, in F3-VP IPDMV subscale the highest level of rejection was found in the 25–49 age group (*p* < .001 in all pairwise comparisons), and in the 18–24 age group the rejection was higher than in the 16–17 one (*p* < .001). Fig 3 shows these patterns of differences by age.

In order to analyze differences by gender in each of the subscales, for each of the four age ranges, a series of subsequent Mann-Whitney tests were conducted, adjusting the levels of significance to control type I error. As shown in Table 5, there were significant differences by gender in the 16–17, 18–24 and 25–49 age groups, and all the differences were in the same sense: women expressed a stronger rejection towards IPVAW than men.

**Table 5:** Differences in attitude dimensions by gender across age groups.

Age	Subscale	Gender (*n*)	*M* (*SD*)	Mean Rank	*U*	*Z*	*p*^a^
**16–17**	**IBIPV**						
	**F1-JVP**	**Men (*n* = 53)**	1.60 (0.82)	66.87	908.000	-3.944	< .001
		**Women (*n* = 57)**	1.21 (0.52)	44.93			
	**F2-VRV**	**Men (*n* = 52)**	1.50 (0.76)	61.55	881.500	-3.334	.001
		**Women (*n* = 52)**	1.14 (0.28)	43.45			
	**F-3ARV**	**Men (*n* = 51)**	3.81 (1.76)	62.45	844.000	-3.307	.001
		**Women (*n* = 53)**	2.72 (1.62)	42.92			
	**IPDMV**						
	**F1-IW**	**Men (*n* = 55)**	1.17 (0.30)	67.71	1006.000	-4.109	< .001
		**Women (*n* = 58)**	1.04 (0.13)	46.84			
	**F3-VP**	**Men (*n* = 54)**	1.84 (0.59)	66.16	936.500	-3.472	.001
		**Women (*n* = 56)**	1.49 (0.46)	45.22			
**18–24**	**IBIPV**						
	**F1-JVP**	**Men (*n* = 173)**	1.27 (0.59)	357.14	28521.500	-6.919	< .001
		**Women (*n* = 435)**	1.07 (0.27)	283.57			
	**F2-VRV**	**Men (*n* = 172)**	1.24 (0.54)	364.89	27969.000	-7.013	< .001
		**Women (*n* = 441)**	1.05 (0.16)	284.42			
	**F-3ARV**	**Men (*n* = 168)**	2.84 (1.85)	354.11	27785.500	-4.611	< .001
		**Women (*n* = 435)**	2.12 (1.43)	281.87			
	**IPDMV**						
	**F1-IW**	**Men (*n* = 173)**	1.09 (0.28)	344.70	31710.500	-5.568	< .001
		**Women (*n* = 441)**	1.02 (0.11)	292.91			
	**F2-BW**	**Men (*n* = 168)**	1.56 (.41)	345.91	28659.500	-4.044	< .001
		**Women (*n* = 432)**	1.41 (0.31)	282.84			
	**F3-VP**	**Men (*n* = 171)**	1.59 (0.55)	346.47	30186.500	-3.753	< .001
		**Women (*n* = 437)**	1.40 (0.41)	288.08			
**25–49**	**IBIPV**						
	**F2-VRV**	**Men (*n* = 23)**	1.16 (0.26)	51.20	547.500	-2.612	.009
		**Women (*n* = 63)**	1.05 (0.20)	40.69			

IBIPV: F1-JPV: *Justifying Partner Violence*; F2-VRV: *Victims Responsible for Violence*; F3-ARV: *Abuser Responsible for Violence*

IPDMV: F1-IW: *Inferiority of Women Compared to Men*; F2-BW: *Blaming Female Victims of Abuse*; F3-VP: V*iolence as an Appropriate Problem-solving Strategy*; F4-MA: *Minimization and Exoneration of the Abuser*.

^a^ Only data from subscales with significant differences are displayed.

Specifically, differences by gender were detected in the 16–17 age group regarding the F1-JPV, F2-VRV, and F3-ARV IBIPV subscales, and the F1-IW and F3-VP IPDMV subscales. In the 18–24 age group we found differences in all of the subscales, except in the F4-MA IPDMV subscale. Finally, in the 25–49 age group there were only differences between men and women in F2-VRV IBIPV subscale (Table 5).

#### Effects of specific IPVAW-training by gender

Given the different number of men and women who had studied some topic related to IPVAW (*n*_men_ = 115; *n*_women_ = 307) and participated in non-curricular activities related to it (*n*_men_ = 126; *n*_women_ = 313) it was advisable to use nonparametric tests to compare the differential effects by gender of these kinds of IPVAW-training. To this end, four groups were established by combining the variable *gender* and the variable *topic* (“yes” vs. “no” studied) on the one hand, and the variables *gender* and *activity* (“yes” vs. “no” participated) on the other hand, followed respectively by a Kruskal-Wallis test to compare each subscale’s scores across groups.

**Fig 4:**
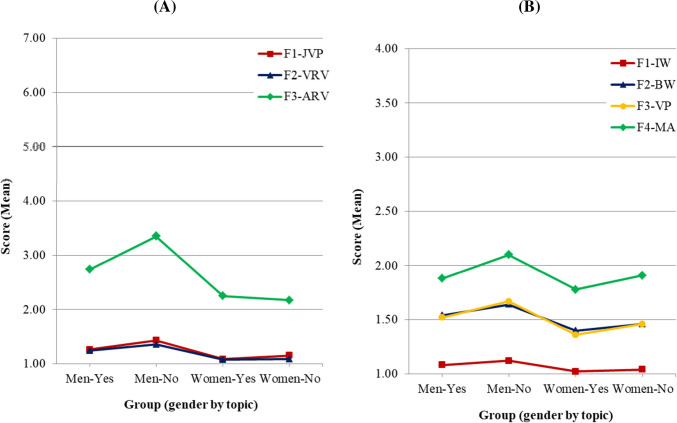
Mean scores of attitude dimensions of men and women having vs. having not studied some topic related to IPVAW. (A) IBIPV: F1-JPV: Justifying Partner Violence; F2-VRV: Victims Responsible for Violence; F3-ARV: Abuser Responsible for Violence. (B) IPDMV: F1-IW: Inferiority of Women Compared to Men; F2-BW: Blaming Female Victims of Abuse; F3-VP: Violence as an Appropriate Problem-solving Strategy; F4-MA: Minimization and Exoneration of the Abuser.

*Effects of having studied topics related to IPVAW*. The Kruskal-Wallis test showed statistically significant differences across groups in all of the subscales of both IBIPV and IPDMV (Table 6). Regarding IBIPV, pairwise comparisons with Bonferroni correction yielded the following results: First, there were differences between men that had studied some topic and those who had not in F1-JPV (*p* = .033), F2-VRV (*p* = .012) and F3-ARV (*p* = .018). In all of these subscales the level of rejection towards IPVAW was higher for those men who had studied some topic, and the largest difference was in the F3-ARV subscale, as shown in Fig 4. No differences were found between women who had studied some topic and women who had not, either in F1-JPV (*p* = .687) or in F2-VRV and F3-ARV (*p* = 1.0 for both subscales).

**Table 6:** Differences in attitude dimensions by having vs. having not studied some topic.

Subscale	Group		*M* (*SD*)	Mean Rank	*H (df = 3)*	*p*
	Gender	Topic (*n*)				
**IBIPV**						
**F1-JVP**	**Men**	**Yes (*n* = 114)**	1.26 (0.60)	481.50	90.657	< .001
		**No (*n* = 159)**	1.43 (0.72)	544.49		
	**Women**	**Yes (*n* = 300)**	1.08 (0.35)	385.02		
		**No (*n* = 296)**	1.15 (0.46)	408.94		
**F2-VRV**	**Men**	**Yes (*n* = 113)**	1.24 (0.62)	473.94	83.816	< .001
		**No (*n* = 158)**	1.35 (0.66)	546.52		
	**Women**	**Yes (*n* = 305)**	1.07 (0.39)	388.16		
		**No (*n* = 291)**	1.08 (0.21)	405.44		
**F3-ARV**	**Men**	**Yes (*n* = 112)**	2.74 (1.85)	456.00	54.131	< .001
		**No (*n* = 151)**	3.35 (1.93)	545.82		
	**Women**	**Yes (*n* = 295)**	2.25 (1.51)	399.55		
		**No (*n* = 291)**	2.17 (1.49)	376.18		
**IPDMV**						
**F1-IW**	**Men**	**Yes (*n* = 115)**	1.08 (0.30)	451.67	67.675	< .001
		**No (*n* = 161)**	1.12 (0.25)	528.40		
	**Women**	**Yes (*n* = 305)**	1.02 (0.09)	399.53		
		**No (*n* = 298)**	1.04 (0.15)	429.16		
**F2-BW**	**Men**	**Yes (*n* = 114)**	1.54 (0.40)	459.52	33.680	**< .001**
		**No (*n* = 154)**	1.64 (0.45)	510.02		
	**Women**	**Yes (*n* = 296)**	1.40 (0.31)	375.74		
		**No (*n* = 285)**	1.46 (0.33)	416.42		
**F3-VP**	**Men**	**Yes (*n* = 114)**	1.52 (0.50)	464.69	33.369	**< .001**
		**No (*n* = 157)**	1.67 (0.61)	512.89		
	**Women**	**Yes (*n* = 303)**	1.36 (0.39)	378.53		
		**No (*n* = 294)**	1.46 (0.43)	438.62		
**F4-MA**	**Men**	**Yes (*n* = 111)**	1.88 (0.55)	421.93	28.800	**< .001**
		**No (*n* = 157)**	2.10 (0.63)	505.42		
	**Women**	**Yes (*n* = 301)**	1.78 (0.52)	378.02		
		**No (*n* = 285)**	1.91 (0.54)	439.00		

IBIPV: F1-JPV: *Justifying Partner Violence*; F2-VRV: *Victims Responsible for Violence*; F3-ARV: *Abuser Responsible for Violence*

IPDMV: F1-IW: *Inferiority of Women Compared to Men*; F2-BW: *Blaming Female Victims of Abuse*; F3-VP: V*iolence as an Appropriate Problem-solving Strategy*; F4-MA: *Minimization and Exoneration of the Abuser*

The pairwise comparisons with IPDMV showed statistically significant differences between men who had studied some topic and men who had not in F1-IW (*p* = .001) and F4-MA (*p* = .036) subscales. There were also differences between women who had studied some topic and women who had not in F3-VP (*p* = .017) and in F4-MA (*p* = .016). In all cases the sense of such differences was the same for both women and men: the rejection towards IPVAW was higher for those who had studied some topic (Table 6 and Fig 4). The largest differences were observed between the two groups of men, and particularly in the F4-MA subscale.

**Fig 5:**
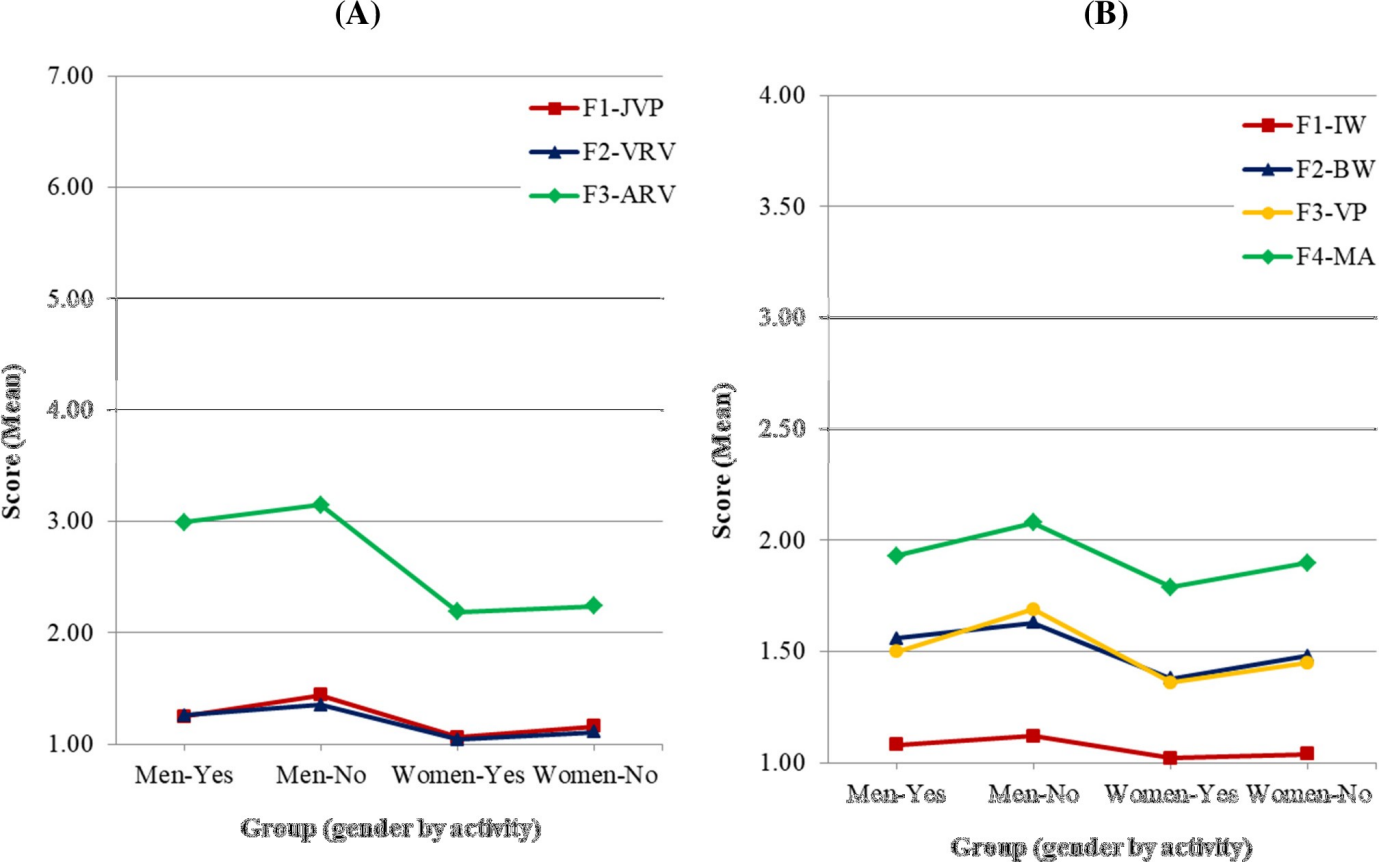
Mean scores of attitude dimensions of men and women having vs. having not participated in non-curricular activities related to IPVAW. (A) IBIPV: F1-JPV: Justifying Partner Violence; F2-VRV: Victims Responsible for Violence; F3-ARV: Abuser Responsible for Violence. (B) IPDMV: F1-IW: Inferiority of Women Compared to Men; F2-BW: Blaming Female Victims of Abuse; F3-VP: Violence as an Appropriate Problem-solving Strategy; F4-MA: Minimization and Exoneration of the Abuser.

*Effects of having participated in non-curricular activities related to IPVAW*. The Kruskal-Wallis test yielded statistically significant differences across groups in both instruments (Table 7). The pairwise comparisons with IBIPV subscales, once the Bonferroni correction was applied, showed a difference in F1-JPV between men who had participated in at least one non-curricular activity and the men who had not (*p* = .036), with higher rejection towards IPVAW in the former. No specific participation effects were found in the women’s sample, either in F1-JPV (*p* = .354) or in F2-VRV and F3-ARV (*p* = 1.0 for both subscales). These results are shown in Fig 5.

**Table 7:** Differences in attitude dimensions by participation in non-curricular activities.

Subscale	Group		*M* (*SD*)	Mean Rank	*H (df = 3)*	*p*
	Gender	Activity (*n*)				
**IBIPV**						
**F1-JVP**	**Men**	**Yes (*n* = 122)**	1.25 (0.49)	486.23	92.674	< .001
		**No (*n* = 153)**	1.44 (0.78)	548.32		
	**Women**	**Yes (*n* = 305)**	1.06 (0.24)	384.40		
		**No (*n* = 293)**	1.16 (0.53)	413.12		
**F2-VRV**	**Men**	**Yes (*n* = 124)**	1.26 (0.54)	495.79	77.385	< .001
		**No (*n* = 149)**	1.35 (0.72)	537.19		
	**Women**	**Yes (*n* = 308)**	1.04 (0.12)	390.01		
		**No (*n* = 290)**	1.11 (0.43)	407.28		
**F3-ARV**	**Men**	**Yes (*n* = 120)**	2.99 (1.87)	501.73	43.494	< .001
		**No (*n* = 145)**	3.15 (1.96)	514.84		
	**Women**	**Yes (*n* = 302)**	2.19 (1.48)	388.18		
		**No (*n* = 286)**	2.24 (1.54)	392.10		
**IPDMV**						
**F1-IW**	**Men**	**Yes (*n* = 125)**	1.08 (0.22)	475.36	58.269	< .001
		**No (*n* = 153)**	1.12 (0.30)	519.86		
	**Women**	**Yes (*n* = 311)**	1.02 (0.10)	404.85		
		**No (*n* = 294)**	1.04 (0.14)	426.60		
**F2-BW**	**Men**	**Yes (*n* = 122)**	1.56 (0.44)	463.28	42.329	< .001
		**No (*n* = 148)**	1.63 (0.43)	512.29		
	**Women**	**Yes (*n* = 296)**	1.38 (0.31)	361.59		
		**No (*n* = 287)**	1.48 (0.33)	435.06		
**F3-VP**	**Men**	**Yes (*n* = 122)**	1.50 (0.54)	441.26	42.283	< .001
		**No (*n* = 151)**	1.69 (0.58)	536.84		
	**Women**	**Yes (*n* = 306)**	1.36 (0.41)	378.01		
		**No (*n* = 291)**	1.45 (0.42)	440.95		
**F4-MA**	**Men**	**Yes (*n* = 121)**	1.93 (0.65)	429.62	26.611	< .001
		**No (*n* = 149)**	2.08 (0.57)	509.26		
	**Women**	**Yes (*n* = 303)**	1.79 (0.53)	383.09		
		**No (*n* = 284)**	1.90 (0.53)	435.61		

IBIPV: F1-JPV: *Justifying Partner Violence*; F2-VRV: *Victims Responsible for Violence*; F3-ARV: *Abuser Responsible for Violence*

IPDMV: F1-IW: *Inferiority of Women Compared to Men*; F2-BW: *Blaming Female Victims of Abuse*; F3-VP: V*iolence as an Appropriate Problem-solving Strategy*; F4-MA: *Minimization and Exoneration of the Abuser*

Regarding the IPDMV, pairwise comparisons showed statistically significant differences between men who had participated in at least one non-curricular activity and men who had not in F3-VP (*p* = .009) and F4-MA (*p* = .049) subscales. Differences were also found between women who had participated in such activities and women who had not in F2-BW (*p* = .002) and F3-VP (*p* = .011) subscales. In all cases the results were the same (Table 7, Fig 5): women and men who had participated in at least one non-curricular activity expressed a stronger rejection towards IPVAW than those who had not.

A subsequent analysis of the relationship between each subscale’s scores and the number of non-curricular activities in which men and women had participated (“one” vs. “from 2 to 5” vs. “more than 5”) was undertaken. The Spearman’s correlation coefficients showed an absence of association between the level of rejection towards IPVAW and the number of activities done by men, for all of the subscales (*p*-values from .188 to .839). However, in the women’s sample a low but significant correlation was found with F3-VP (*r* = -.160, *p* = .005) and F4-MA (*r* = -.152, *p* = .008) IPDMV subscales. In both cases a negative coefficients imply a positive association between the level of rejection towards IPVAW and the number of activities done by women, the higher the participation, the stronger the rejection. The rest of the correlations in women’s sample were statistically null (*p*-values from .156 to .511).

## Discussion

The results obtained make it possible to achieve the objectives proposed for the two studies carried out, that is, to broaden our knowledge of supportive attitudes towards IPVAW, once analyzed and improved the measures tools employed.

The aim of Study 1 was to analyze the internal consistency and factorial structure of two questionnaires, IBIPV and IPDMV, which measure attitudes towards IPVAW and the level of convergence-divergence among their subscales.

The results obtained in this preliminary analysis suggest that the IBIPV factorial structure proposed by their authors [64] may work reasonably well, in general and for F2-VRV and F3-ARV subscales, but not enough for the F1-JPV subscale. Therefore, on the basis of the analysis of the corrected homogeneity indices, we removed four items that negatively affected the internal consistency, one item of the F1-JPV subscale, one item of the F2-VRV subscale, and two items of the F3-ARV subscale. The elimination of these 4 items improved the reliability of their respective subscales and the overall model fit. We may hypothesize that this improvement is due to the format of these items, since item 3 of the Spanish version (“Even though men’s masculinity is threatened when their partner points out their weak points, men don't have the right to beat up their partners”) is the only item in the JPV subscale that scores in an opposite sense, and items 15 (“Battered women should separate from their partners immediately”), 21 (“The best way to combat violence against women is to force the abuser to attend couple’s counselling”) and 22 (“The best way to combat violence against women is to arrest the perpetrators”) break the response pattern of the F2-VRV and F3-ARV subscales, respectively. In fact, this result is not unexpected since García-Ael et al. [64] already found that factor loadings were high, except in the case of items 6 (item 3 in the Spanish version), 15, 21 and 22. In summary, the results obtained confirm the three-factor model proposed by Garcia-Ael et al. [64], suggesting that IBIPV, with the improvement obtained from eliminating the four items aforementioned, may be an adequate tool to assess one of the supportive attitudes towards IPVAW, the legitimation [15, 19, 26], given that their subscales assess the IPVAW justification (F1-JPV), the victim blaming (F2-VRV), and the exoneration of the perpetrator (measured in an opposing sense by the F3-ARV subscale).

Additionally, the results obtained confirm that the four-dimension IPDMV model obtained by Ferrer et al. [61] worked appropriately, although the internal consistency of some of its subscales continues to be its main weakness. In any case, it may be opportune to remember that in basic research studies like the present, reliability values around .50 might be considered as acceptable [72–74], particularly if they are scales with a small number of items [75]. Regarding its content, this tool also assesses the IPVAW legitimation as supportive attitudes towards IPVAW [15, 19, 26], by the F2-BW and F4-MA subscales, and provides a measure of sexist beliefs (by the F1-IW subscale) and of attitudes towards violence in general (by the F3-VP subscale).

The pattern of correlations between scale scores provided evidence of the IBIPV’s validity, based on their relationship with other variables [76]. Thus, the convergence found in aspects related to general justifying IPVAW and perception of women (i.e. inferiority and blaming victims) is theoretically consistent [12, 19–21, 25]. In the same sense, the lower correlations found between IBIPV subscales and the IPMDV F3-VP subscale is also theoretically consistent, in line with previous studies (e.g. [64, 65, 77]), since the latter refers to general justifying of violence (not specifically to IPVAW). Additionally, the absence of a relationship between the F3-ARV IBIPV subscale and the F4-MA IPDMV subscale pointed to the different aspects they measured, having the common factor of the abuser responsibility, but contributing differentially with complementary nuances. Finally, it is also interesting to point out that the IBIPV’s JPV, VRV, and ARV (with scores inverted) subscales were positively and significantly correlated with F1-IW IPDMV subscale, which means that the more sexist the respondents are, the more they tend to justify IPVAW, blaming victims, and exonerate perpetrators [51, 64].

On the other hand, the aim of Study 2 was to analyze the attitudes towards IPVAW across dimensions, and to compare them by gender, age, and specific training in IPVAW.

First of all, we evaluated differences between subscale scores separately for men and women. The results obtained showed statistically significant differences in IBIPV and in IPDMV, and for both men and for women. Regarding IBIPV, the pairwise comparisons showed that in the women’s sample there were significant differences between all subscales, with the strongest rejection towards IPVAW in F2-VRV, and the lowest in F3-ARV; and in the men’s sample there were significant differences between F1-JPV and F3-ARV, and between F2-VRV and F3-ARV (but not between F1-JPV and F2-VRV). Regarding IPDMV, the pairwise comparisons showed the same pattern in both subscales: all comparisons were significant, except between F2-BW and F3-VP subscales; and the strongest rejection towards IPVAW was done in F1-IW, and the lowest in F4-MA.

Secondly, the comparison by gender in each of the IBIPV and IPDMV subscales showed a gender gap, as expected [19, 23–27, 29, 31, 43]. Thus, women expressed a stronger rejection towards IPVAW in all cases, which is consistent with previous research [16, 43, 47, 50, 60, 61, 63, 64].

In summary, we can say that both men and women are samples with high rejection towards IPVAW, particularly towards beliefs about IPVAW justification and blaming victims; in contrast, beliefs about perpetrators exoneration generate more acquiescence.

Next we studied the differences between age groups. The results obtained reflected an age effect in the sense that the youngest and the oldest people (16–17 years old and 50–82 years old groups) showed a lower rejection towards IPVAW (F1-JPV IBIPV subscale) and towards sexist attitudes (F1-IW IPDMV subscale), and a stronger acquiescence towards beliefs exonerating perpetrators (F4-MA IPMDV subscale). Additionally, the youngest people showed the lowest rejection towards the responsibility of perpetrators (F3-ARV IBIPV subscale), and towards blaming victim (F2-VRV IBIPV and F2-BW IPDMV subscales) in a level similar to the oldest people. As Flood and Pease [19] pointed out, the best results in young people and young adults (between 18 to 49 years old) might be expected, and could be explained by the improvements over time in attitudes towards women and against VAW, by their exposure to university, and by other positive informative influences. In turn, older generations would tend to hold more traditional views regarding the acceptance of violence as a normal part of intimate partner relationships, as IPVAW was not considered a social problem when they reached their adulthood [40]. In summary, these results suggest a U-form relation between age and supportive attitudes towards IPVAW, congruent with previous research results [26, 29, 35, 40, 52, 53]. This U-effect might explain the age effect inconsistences pointed out by some authors [15, 27, 43, 46, 50]. A really worrying result concerns attitudes observed in the youngest population. In this sense, the IPVAW supportive attitudes showed by the youngest people may reflect [19, 25, 35]: their lack of exposure to the influence of higher levels of education; developmental shifts in attitudes and qualities, such as empathy, sensitivity, or moral awareness; or distinct characteristics of peer culture (particularly, boys’ peer culture, given that gender segregation and homophobia peak in early boys adolescence). These circumstances point out that challenging and changing attitudes that tolerate and justify IPVAW are really a key objective for their prevention and eradication [10, 15], and it is most notable among young boys.

The differences in IPVAW supportive attitudes by gender across age groups showed that there were significant differences by gender in sexist attitudes, IPVAW justification, blaming victims, and exonerating perpetrators, but essentially only in the youngest groups (16–17 and 18–24 years old), and all these differences were in the same sense: women expressed a stronger rejection towards IPVAW than men. These results suggest the need for further studies on the effects of age and other related factors (such as, educational level) on gender gap [25].

Finally, we studied the differences between people who had received previous IPVAW training and those who had not. In general, the results obtained reflected a training effect, in a sense similar to that observed in previous research; i.e., people who had received IPVAW training showed less supportive attitudes towards it [60, 61]. However, it is important to note that in previous research these IPVAW-training effects occurred mainly among women [60], while the current results showed effects on both men and women, although they are slightly different depending on the assessment instrument applied.

Thus, the IBIPV results exhibited a positive effect of IPVAW-training on attitudes only among men, so that those who had studied some topic related to IPVAW showed less supportive attitudes to it, especially less justification of the perpetrators (F3-ARV), and those who had participated in some non-curricular activity were less likely to justify this violence (F1-JPV). A possible explanation is that IBIPV focuses specifically on supportive attitudes towards IPVAW that already have a very high level of rejection among women, leaving little scope for change, and therefore for appreciating the training effects among them.

In contrast, the IPDMV results displayed positive effects of IPVAW-training on attitudes in both men and women. Thus, among men, those who had studied some topic related to IPVAW showed less sexist attitudes (F1-IW), those who had carried out some non-curricular activity related to IPVAW were less likely to justify the use of violence as a general strategy (F3-VP), and those who had received one of the two forms of IPVAW-training were less likely to exonerate the aggressors (F4-MA). Among women, those who had had studied some topic exonerated the aggressors less (F4-MA), those who had carried out some non-curricular activity blamed the victim less (F2-BW), and those who had received some form of IPVAW-training were less likely to justify the use of violence as a general strategy (F3-VP), and, furthermore, among women, the more activities they had carried out, the less favorable were the attitudes they exhibited towards the use of violence as a general strategy (F3-VP) and towards exonerating abusers (F4-MA). These results therefore give additional information on IPVAW-training effects on general attitudes, such as sexist beliefs or justification of the use of violence.

In short, the results obtained have implications for future research and practice in IPVAW because they provide useful information about some measure tools for attitudes towards this violence. Concretely, these results obtained that both IBIPV and IPDMV may be useful questionnaires for evaluating supporting IPVAW attitudes in Spanish-speaking samples, and provide complementary information about them. Thus, IBIPV is more focused on these supporting attitudes and seems more effective for capturing differences between men and women in blaming victims and exonerating perpetrators. IPDMV includes additional information regarding the minimization of IPVAW and the responsibility of perpetrators; it seems better able to capture the effect of specific IPVAW-training, which would be consistent with the fact that it was initially designed to detect the effects of interventions [57, 58], although it has subsequently been found to have a low sensitivity to therapeutic change and a capacity to discriminate between samples [62]. In addition, IPDMV complements the information on specific attitudes towards IPVAW with general information, equally related to IPVAW [20, 21, 25], such as sexist beliefs, and the use of violence in general. Additionally, these results allow us to complement previous studies on the effects that factors such as gender, age, or previous training have on supportive attitudes towards IPVAW [60, 61, 64].

Although this study makes some contributions to the field, it is not without limitations. The main limitation is the use of an opportunity sample where population heterogeneity in certain variables was not adequately represented, that is to say women, people with higher education, students with previous training in IPVAW, and people with high a level IPVAW rejection were over-represented. This composition means that the results obtained cannot be generalized to the population, especially to groups with a lower level of education since, as Wang [25] points out, the educational level may be a fundamental modulating factor in attitudes towards IPVAW. Furthermore, this decompensation in the composition of the sample meant that the size of the subsamples formed according to the different variables was very unequal, which made it necessary to carry out non-parametric analyses, limiting the use of other statistical techniques. An additional limitation has to do with the low internal consistency of some of the subscales of the questionnaires used (particularly, F1-JPV of IBIPV, and F4-MA of IPDMV).

A strength of this study is that, unlike many studies on attitudes towards IPVAW (i.e., [44, 63]), it not only includes young people (under 25 years), but also includes adults and older adults (17.3% are between 25 and 82 years old). However, as this proportion was small, it was not possible to analyze the effect of IPVAW-training by gender in different age groups. Further research using larger, more diverse and probabilistic samples may be helpful in overcoming these limitations, as well as incorporating other variables described in the literature on the topic, to function as possible modulators of supportive attitudes to IPVAW [19, 25].

## References

[1] Devries KM, Mak JYT, Garcia-Moreno C, Petzold M, Child JC, Felder G, et al The global prevalence of intimate partner violence against women. Science. 2013; 340: 1527–1528. 10.1126/science.1240937 23788730

[2] Ellsberg M, Jansen HAFM, Heise LL, Watts CH, Garcia-Moreno C on behalf of the WHO Multi-country Study on Women’s Health and Domestic Violence against Women Study Team. Intimate partner violence and women’s physical and mental health in the WHO multi-country study on women’s health and domestic violence: an observational study. The Lancet. 2008; 371(9619): 1165–1172. 10.1016/S0140-6736(08)60522-X 18395577

[3] Garcia-Moreno C, Jansen HA, Ellsberg M, Heise LL, Watts CH. Prevalence of intimate partner violence: findings from the WHO multi-country study on women’s health and domestic violence. The Lancet. 2006; 368(9543): 1260–1269. 10.1016/S0140-6736(06)69523-17027732

[4] Stockl H, Devries K, Rotstein A, Abrahams N, Campbell J, Watts C, et al The global prevalence of intimate partner homicide: a systematic review. The Lancet. 2013; 382: 859–865. 10.1016/S0140-6736(13)61030-2 23791474

[5] World Health Organization. Global and regional estimates of violence against women: Prevalence and health effects of intimate partner violence and non-partner sexual violence. Geneva, Switzertland: WHO; 2013.

[6] EUFRA (European Union Agency for Fundamental Rights). Violence against women: An EU-wide survey. Luxembourg: Publications Office of the European Union; 2014 Available from: http://fra.europa.eu/en/publication/2014/vaw-survey-main-results_en.pdf

[7] UN (United Nations). Declaration on the Elimination of Violence against Women (A/RES/48/104). NY: UN; 1994 Available from: http://www.un.org/documents/ga/res/48/a48r104.htm

[8] Istanbul Convention. Council of Europe Convention on preventing and combatting violence against women and domestic violence. Istanbul, Turkey: Council of Europe Committee of Ministers; 2011.

[9] EIGE (European Institute for Gender Equility). Glossary & Thesaurus. Vilnius: EIGE; 2020 Available from: https://eige.europa.eu/thesaurus/terms/1265?lang = en

[10] Garcia-Moreno C, Zimmerman C, Morris-Gehring A, Heise LL, Amin A, Abrahams N, et al Addressing violence against women: a call to action. The Lancet. 2015; 385: 1685–1695. 10.1016/S0140-6736(14)61830-4 25467579

[11] Gracia E. Intimate partner violence against women and victim-blaming attitudes among European. Bull World Health Organ. 2014; 92: 380–381. 10.2471/BLT.13.131391 24839328 PMC4007130

[12] Gracia E, Lila M. Attitudes towards violence against women in the EU. Luxembourg: Publications Office of the European Union; 2015.

[13] Kury H, Obergfell-Fuchs J, Woessner G. The extent of family violence in Europe: a comparison of Nacional Surverys. Violence Against Women. 2004; 10: 749–769. 10.1177/1077801204265550

[14] Nayak MB, Byrne CA, Martín MK, Abraham AG. Attitudes toward violence against women: a cross-nation study. Sex Roles. 2003; 49: 333–342. 10.1023/A:1025108103617

[15] Gracia E, Lila M, Santirso FA. Attitudes toward intimate partner violence against women in the european union: a systematic review. European Psychologist. 2020 First online. 10.1027/1016-9040/a000392

[16] Arnoso A, Ibabe I, Arnoso M, Elgorriaga E. El sexismo como predictor de la violencia de pareja en un contexto multicultural [Sexism as predictor of intimate partner violence in a multicultural context]. Anuario de Psicología Jurídica. 2017; 27: 9–20. 10.1016/j.apj.2017.02.001

[17] Capaldi DM, Knoble NB, Shortt JW, Kim HK. A systematic review of risk factors for intimate partner violence. Partner Abuse. 2012; 3(2): 231–280. 10.1891/1946-6560.3.2.231 22754606 PMC3384540

[18] Flood M, Pease B. Rethinking the significance of attitudes in preventing men's violence against women. Australian Journal of Social Issues. 2008; 43: 547–561. 10.1002/j.1839-4655.2008.tb00118.x

[19] Flood M, Pease B. Factors influencing attitudes to violence against women. Trauma, Violence Abuse. 2009; 10: 125–142. 10.1177/1524838009334131 19383630

[20] Heise LL, Kotsadam A. Cross-national and multilevel correlates of partner violence: an analysis of data from population-based surveys. Lancet Glob Health. 2015; 3: e332–340. 10.1016/S2214-109X(15)00013-3 26001577

[21] Puente A, Ubillos S, Echeburua E, Paez D. Risk factors associated with the violence against women in couples: a review of meta-analysis and recent studies. Anales de Psicología. 2016; 32(1): 295–306. 10.6018/analesps.32.1.189161

[22] Romero A, Lila M, Gracia E, Rodríguez CM, Moya L. Acceptability if intimate partner violence among male offenders: the role of set-shifting and emotion decoding dysfuntions as cognitive risk factors. Int J Environ Res Public Health. 2019; 16(9): 1537 10.3390/ijerph16091 PMC6539109 31052264

[23] Sardinha L, Najera HE. Attitudes towards domestic violence in 49 low- and middle-income countries: A gendered analysis of prevalence and country-level correlates. PLoS One. 2018; 13(10): e0206101 10.1371/journal.pone.0206101 30379891 PMC6209205

[24] Stith SM, Smith DB, Penn CE, Ward DB, Tritt D. Intimate partner physical abuse perpetration and victimization risk factors: A meta-analytic review. Aggression and Violent Behavior, 2004; 10, 65–98. 10.1016/j.avb.2003.09.001

[25] Wang L. Factors influencing attitude toward intimate partner violence. Aggression and Violent Behavior. 2016; 29: 72–78. 10.1016/j.avb.2016.06.005

[26] Gracia E, Rodríguez CM, Lila M. Preliminary evaluation of an analogue procedure to assess acceptability of intimate partner violence against women: the Partner Violence Acceptability Movie Task. Frontiers in Psychology. 2015; 6: 1567 10.3389/fpsyg.2015.01567 26528220 PMC4600898

[27] Bucheli M, Rossi M. Attitudes toward intimate partner violence against women in Latin America and the Caribbean. Criminology and Criminal Justice. 2019; 9(3): 1–13. 10.1177/2158244019871061

[28] Herrero J, Torres A, Rodríguez FJ, Juarros-Basterretxea J. Intimate partner violence against women in the European Union: the influence of male partners’ traditional gender roles and general violence. Psychology of Violence. 2017; 7(3): 385–394. 10.1037/vio0000099

[29] Ivert AK, Merlo J, Gracia E. Country of residence, gender equality and victim blaming attitudes about partner violence: a multilevel analysis in EU. Eur J Public Health. 2018; 28: 559–564. 10.1093/eurpub/ckx138 29036678

[30] Tausch A. Multivariate analyses of the global acceptability rates of male intimate partner violence (IPV) against women based on World Values Survey data. Int J Health Plann Manage. 2019; 34: 1155–1194. 10.1002/hpm.2781 30977561

[31] Willis C, Delgado RH. Attitudes toward violence and gender as predictors of interpersonal violence interventions. J Interpers Violence; 2020; 35(3–4): 809–827. 10.1177/0886260517690872 29294644

[32] European Commission. Europeans and their views on domestic violence. Eurobarometer 51.0. European Commission. Directorate-General X “Information, Communication, Culture and Audiovisual Media”. Brussels, Belgium: Author; 1999 Available from: http://ec.europa.eu/public_opinion/archives/ebs/ebs_127_en.pdf

[33] European Commission. Domestic violence against women. Special Eurobarometer 73.2. Brussels, Belgium: TNS Opinión & Social; 2010 Available from: http://ec.europa.eu/public_opinion/archives/ebs/ebs_344_en.pdf

[34] Gracia E, Herrero J. Acceptability of domestic violence against women in the European Union: A multilevel analysis. J Epidemiol Community Health. 2006; 60: 123–129. 10.1136/jech.2005.036533 16415260 PMC2588066

[35] Waltermaurer E. Public justification of intimate partner violence: a review of the literature. Trauma Violence Abuse. 2012; 13: 167–175. 10.1177/1524838012447699 22643069

[36] Ferrer VA, Bosch E. Gender violence as a social problem in Spain: Attitudes and acceptability. Sex Roles. 2014; 70(11–12): 506–521. 10.1007/s11199-013-0322-z

[37] Meil G. Percepción social de la violencia de género [Social perception of gender violence]. Madrid: Ministerio de Sanidad, Servicios Sociales e Igualdad. Centro de Publicaciones; 2014.

[38] Spanish Government Office for Gender-Based Violence. Análisis de la encuesta sobre percepción social de la violencia de género [Analysis of the survey on social perception of gender violence]. Madrid: Ministerio de Sanidad, Servicios Sociales e Igualdad; 2014.

[39] Spanish Ministry of Health, Social Services and Equality. Percepción social de la violencia de género [Social perception of gender violence]. Colección contra la violencia de Género. Madrid: Ministerio de Sanidad, Asuntos Sociales e Igualdad. Centro de Publicaciones; 2013.

[40] Gracia E, Tomás JM. Correlates of victim-blaming attitudes regarding partner violence against women among the Spanish general population. Violence Against Women. 2014; 20(1): 26–41. 10.1177/1077801213520577 24476756

[41] Abramsky T, Watts CH, Garcia-Moreno C, Devries K, Kiss L, Jansen HAFM, et al What factors are associated with recent intimate partner violence? Findings from the WHO multi-country study on women’s health and domestic violence. BMC Public Health. 2011; 11(109): 1–17. 21324186 10.1186/1471-2458-11-109 PMC3049145

[42] Berkel LA, Vandiver BJ, Bahner AD. Gender role attitudes, religion and spirituality as predictors of domestic violence attitudes in white college students. Journal of College Student Development. 2004; 45: 119–133. 10.1353/csd.2004.0019

[43] Martín-Fernández M, Gracia E, Marco M, Vargas V, Santirso FA, Lila M. Measuring acceptability of intimate partner violence against women: development and validation of the A-IPVAW Scale. The European Journal of Psychology Applied to Legal Context. 2018; 10(1): 26–34. 10.5093/ejpalc2018a3

[44] Ferragut M, Blanca MJ, Ortiz-Tallo M, Bendayan R. Sexist attitudes and beliefs during adolescence: A longitudinal study of gender differences. European Journal of Developmental Psychology. 2017; 14(1): 32–43. 10.1080/17405629.2016.1144508

[45] Archer J, Graham-Kevan N. Do beliefs about aggression predict physical aggression to partners? Aggressive Behavior. 2003; 29: 41–54. 10.1002/ab.10029

[46] Megías JL, Toro V, Carretero H. The acceptance of myths about intimate partner violence against women (AMIVAW) scale: development and validation in Spanish and English. Psychology of Women Quarterly. 2018; 42(1): 44–61. 10.1177/0361684317742638

[47] Vidal-Fernández A, Megías JL. Attributions of blame to battered women when they are perceived as feminists or as “difficult to deal with”. Span J Psychol. 2014; 17: E. 21. 10.1017/sjp.2014.26 25011491

[48] Alfredsson H, Ask K, von Borgstede C. Motivational and cognitive predictors of the propensity to intervene against intimate partner violence. J Interpers Violence. 2014; 29: 1877–1893. 10.1177/0886260513511696 24366962

[49] Cinquegrana V, Baldry AC, Pagliaro S. Intimate partner violence and bystanders’ helping behaviour: An experimental study. Journal of Aggression, Conflict and Peace Research. 2018; 10: 24–35. 10.1108/JACPR-08-2016-0243

[50] Martín-Fernández M, Gracia E, Lila M. Assessing victim-blaming attitudes in cases of intimate partner violence against women: Development and validation of the VB-IPVAW scale. Psychosocial Intervention. 2018; 27: 133–143. 10.3389/fpsyg.2018.01146 30065678 PMC6056762

[51] Valor-Segura I, Expósito F, Moya M. Victim blaming and exoneration of the perpetrator in domestic violence: The role of beliefs in a just world and ambivalent sexism. Span J Psychol. 2011; 14: 195–206. 10.5209/rev_SJOP.2011.v14.n1.17 21568177

[52] Correia I, Alves H, Morais R, Ramos M. The legitimation of wife abuse among women: The impact of belief in a just world and gender identification. Personality and Individual Differences. 2015; 76: 7–12. 10.1016/j.paid.2014.11.041

[53] Gracia E, García F, Lila M. Public responses to intimate partner violence against women: The influence of perceived severity and personal responsibility. Span J Psychol. 2009; 12: 648–656. 10.1017/s1138741600002018 19899665

[54] Delgado C, Estrada B, López JA. Gender and cultural effects on perception of psychological violence in the partner. Psicothema. 2015; 27(4): 381–387. 10.7334/psicothema2015.54 26493577

[55] Villegas G, González N, Sánchez-García AB, Sánchez M, Galindo-Villardón MP. Seven methods to determine the dimensionality of tests: application to the General Self-Efficacy Scale in twenty-six countries. Psicothema.2018; 30(4): 442–448. 10.7334/psicothema2018.113 30353847

[56] López-Cepero J, Rodríguez L, Rodríguez FJ. Evaluación de la violencia de pareja. Una revisión de instrumentos de evaluación conductual [Assessment of partner violence. A review of behavioural assessment instruments]. Revista Iberoamericana de Diagnóstico y Evaluación–e Avaliação Psicológica. 2015; 2(40): 37–50.

[57] Echeburúa E, Fernández-Montalvo J. Tratamiento cognitivo-conductual de hombres violentos en el hogar: un estudio piloto [Cognitive-behavioral treatment of violent men in the home: a pilot study]. Análisis y Modificación de Conducta. 1997; 23(89): 355–384.

[58] Echeburúa E, Fernández-Montalvo J (1998). Hombres maltratadores. Aspectos teóricos [Batterer men. Theoretical aspects] In Echeburúa E, Corral P editors. Manual de violencia familiar. Madrid: Siglo XXI; 1998 pp. 73–90. 10.1177/01454455980223003

[59] Echeburúa E, Amor PJ, Sarasua B, Zubizarreta I, Holgado-Tello FP. Inventario de Pensamientos Distorsionados sobre la Mujer y el Uso de la Violencia Revisado (IPDMUV-R): propiedades psicométricas [Inventory of Distorted Thoughts about Women and the Use of Violence-Revised (IPDMUV-R): psychometric properties]. Anales de Psicología. 2016; 32(3): 837–846. 10.6018/analesps.32.3.231901

[60] Ferrer VA, Bosch E, Ramis C, Torres G, Navarro C. La violencia contra las mujeres en la pareja: creencias y actitudes en estudiantes universitarios [Violence against women in relationships: beliefs and attitudes in university students]. Psicothema. 2006; 18(3): 359–366. 17296057

[61] Ferrer VA, Bosch E, Sánchez-Prada A, Delgado C. Beliefs and attitudes about intimate partner violence against women in Spain. Psicothema. 2019; 31: 38–45. 10.7334/psicothema2018.206 30664409

[62] Loinaz I. Distorsiones cognitivas en agresores de pareja: análisis de una herramienta de evaluación [Cognitive distortions among partner-violent men: analyzing an assessment tool]. Terapia Psicológica. 2014; 32(1): 5–17. 10.4067/S0718-48082014000100001

[63] Ubillos S, Goiburu E, Puente A, Pizarro JP, Echeburúa E. Assessment of distorted thoughts about women and violence of Basque-speaking secondary school students. Revista de Psicodidáctica. 2017; 22(1): 1–8. 10.1387/RevPsicodidact.16124

[64] García-Ael C, Recio P, Silván-Ferrero P. Psychometric properties of the inventory of beliefs about intimate partner violence (IBIPV). Anales de Psicología. 2018; 34: 135–145. 10.6018/analesps.34.1.232901

[65] Saunders DG, Lynch AB, Grayson M, Linz D. The inventory of beliefs about wife beating: The construction and initial validation of a measure of beliefs and attitudes. Violence Vict. 1987; 2: 39–57. 10.1891/0886-6708.2.1.39 3154156

[66] Forero CG, Maydeu A, Gallardo D. Factor analysis with ordinal indicators: a Monte Carlo study comparing DWLS and ULS estimation. Structural Equation Modeling A Multidisciplinary Journal. 2009; 14(4): 625–641. 10.1080/10705510903203573

[67] Morata MA, Holgado FP, Barbero I, Méndez G. Análisis factorial confirmatorio. Recomendaciones sobre mínimos cuadrados no ponderados en función del error tipo I, de ji cuadrado y RMSEA [Confirmatory factor analysis. Recommendations for unweighted least squares method related to Chi-Square and RMSEA]. Acción Psicológica. 2015; 12(1): 79–90. 10.5944/ap.12.1.14362

[68] Byrne BM. Structural equation modelling with AMOS: basic concepts, applications, and programming (2nd ed.). New York, NY: Routledge; 2010

[69] Hu L, Bentler PM. Cut-off criteria for fit indexes in covariance structure analysis: conventional criteria versus new alternatives. Structural Equation Modeling. 1999; 6(1): 1–55. 10.1080/10705519909540118

[70] Schermelleh-Engel K, Moosbrugger H, Müller H. Evaluating the fit of structural equation models: tests of significance and descriptive goodness-of-fit measures. Methods of Psychological Research Online. 2003; 8(2): 23–74.

[71] Muliak SA, James LR, Van Alstine J, Bennett N, Lind S, Stilwell CD. Evaluation of goodness-of-fit indices for structural equation models. Psychol Bull. 1989; 5(3): 430–455.

[72] Morales P, Urosa B, Blanco A. Construcción de escalas de actitudes tipo Likert [Construction of likert attitude scales] Madrid: La Muralla; 2003.

[73] Nunnally JC. Psychometric Theory. New York: McGraw-Hill; 1967.

[74] Uses Schmitt N. and abuses of coefficient alpha. Psychol Assess. 1996; 8(4): 350–353. 10.1037/1040-3590.8.4.350

[75] Delgado C. Viajando a Ítaca por mares cuantitativos. Manual de ruta para investigar en grado y postgrado [Travelling to Ithaca on quantitative seas Route manual for undergraduate and graduate research]. Salamanca: Amarú; 2014.

[76] AERA, NCME, & APA. Standards for educational and psychological testing. Washington, DC: AERA; 2014.

[77] Sanchez-Prada A, Delgado-Alvarez C, Bosch E, Ferrer VA. Aportaciones sobre la Medición de Creencias acerca del Maltrato a la Mujer (IBWB) en Población Española [Contributions on the Measurement of Beliefs about the Abuse of Women (IBWB) in the Spanish Population]. Revista Iberoamericana de Diagnóstico y Evaluación–e Avaliação Psicológica. 2019; 53(4): 49–62. 10.21865/RIDEP53.4.04

